# A Questionnaire Dataset on Perceived Stress in Indian Higher Education Students during Emergency Remote Learning

**DOI:** 10.12688/f1000research.159192.1

**Published:** 2025-01-07

**Authors:** Sharisha Shanbhog M, Jeevan Medikonda, Shweta Rai, Rayan Charls Mathias

**Affiliations:** 1 Manipal Institute of Technology, Manipal, Karnataka, 576104, India; 2 Manipal Academy of Higher Education, Manipal, Karnataka, 576104, India; 3 Manipal College of Health Professions, Manipal, Karnataka, 5761014, India

**Keywords:** Emergency Remote Learning, Perceived Student Stress, Stress level analysis, Questionnaire data, Visualizations.

## Abstract

**Background:**

The COVID-19 pandemic led to a sudden shift to Emergency Remote Learning, significantly impacting students’ mental health. This study visualizes and analyses various stressors contributing to stress levels among university students during Emergency Remote Learning and explores how different factors from environmental and instructional mediums contribute to their perceived stress.

**Method:**

Data was collected through a cross-sectional survey using the Modified Perceived Stress Scale and an additional set of 20 Likert scale items on Emergency Remote Learning. One-sample t-tests were performed to assess the consistency of responses across questionnaire items, and correlation analysis was conducted to examine the relationships between different stressors. Frequency distributions were also analyzed to capture the prevalence of stress levels across demographic, environmental, and instructional variables.

**Conclusion:**

The study revealed that frequent thoughts about unaccomplished tasks were associated with high stress (14.12%). Other factors include feeling nervous and stressed, feeling things that are not going their way, and difficulties piling up significantly elevated stress levels. Items specific to Emergency Remote Learning revealed that a sudden shift to Remote Learning heavily influenced students’ mental well-being. Additionally, demographic analysis showed that students aged 21 experienced the highest stress levels. Living arrangements, internet connectivity, and the impact of COVID-19 on close affinities further contributed to stress. This study underscores the complexity of stress during Emergency Remote Learning. It emphasizes the need for institutions to address psychological and instructional factors to support students better during remote learning environments.

## Introduction

Stress among students is a significant issue in higher education since it affects their overall quality of life, mental and physical health, and academic performance. ^1, 2^ Among various factors causing stress among students, the stress of academics causes mild to severe depression in 31.64% of Indian Higher Education students. ^3, 4^ Moreover, several components within academia can affect academic stress in higher education, like teaching, social relations, and environmental factors. ^5^

In an already stressful academic environment, the year 2020 and beyond brought unprecedented challenges. Educational institutions worldwide were forced to shut down to restrict the spread of the COVID-19 virus. ^6^ This period of uncertainty caused widespread stress, anxiety, isolation, and a sense of helplessness as individuals faced emotional, psychological, economic, and social crises. ^7^ India, home to a large population of young adults (aged 19-35), faced various challenges, particularly in the academic domain. ^8^ The COVID-19 pandemic thus had significantly increased already existing stress levels among higher education students. A large community of students reported increased levels of stress and anxiety due to health concerns, academic performance, and social isolation during the pandemic. ^9– 12^

One of the most significant disruptions post-COVID-19 pandemic was due to the overnight transition of traditional classroom learning to an online platform ^13, 14^ with many students finding it less effective and more stressful compared to traditional in-person learning. ^15– 17^ This unplanned shift, termed Emergency Remote Learning (ERL), ^18^ was necessary to ensure the continuity of academic activities but came with numerous psychological and logistical challenges. ^19, 20^ Research studies indicate that many students experienced increased levels of stress and anxiety during this period, with the situation worsening as the pandemic progressed. ^21^ Among the affected people, post-pandemic psychological stress was more common among students, especially in health-related disciplines worldwide, with a prevalence rate of 44% in the study group. ^22^

**Figure 1.**
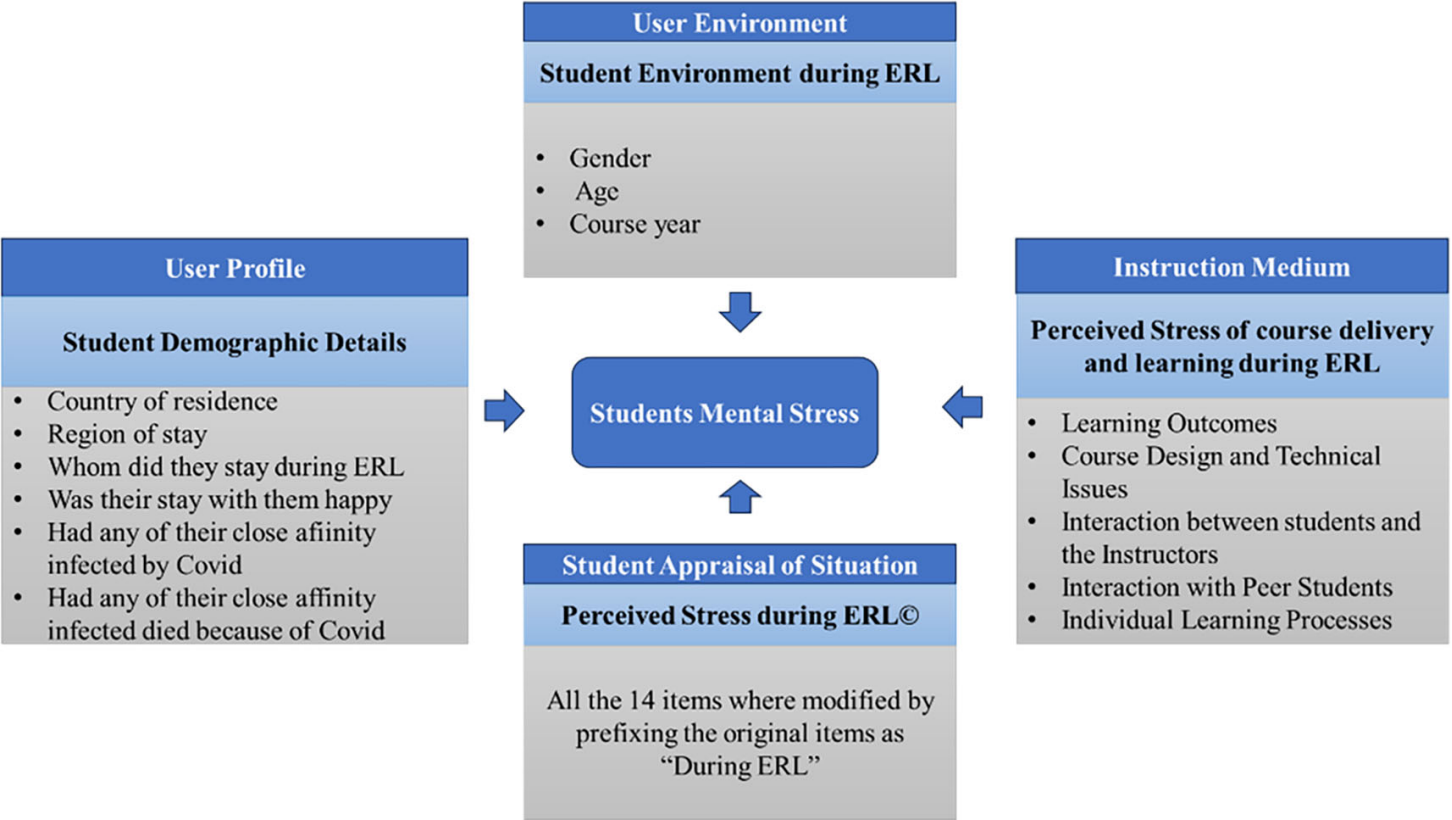
The Overall Structure of the Multidomain Questionnaire.

Since an abrupt shift to Emergency Remote Learning (ERL) has increased the concerns about academic performance, which is a significant stressor ^23, 24^ this study is conducted to identify the various factors contributing to stress during ERL and how these stressors influence the mental well-being of students. A multidomain questionnaire shown in Figure 1 was developed and validated at different stages to assess students’ stress levels in Emergency Remote Learning (ERL) circumstances. While a study exists to analyze factors contributing to stress among faculties during the pandemic ^25^ not much literature exists that studies different stressors contributing to stress among students during the pandemic. Moreover, several standard instruments to measure anxiety, depression, and general perceived stress exist. At the same time, no tool has been developed to analyze students’ stress specifically related to the challenges associated with Emergency Remote Learning with a psychological perspective on student’s mental state. A newly created and validated multidomain questionnaire was thus used to measure stress across various parameters. Data was collected from 1,200 students across different engineering disciplines to get diverse perspectives on the stressors associated with ERL and their cumulative impact on academic performance.

Hence, this questionnaire dataset offers valuable insights into the factors contributing to stress among students during ERL and the broader implications for their mental health. These survey responses from students serve as a resource for researchers, educators, and policymakers interested in understanding and mitigating the psychological impact of remote learning, especially during times of Emergency. By examining the stressors identified in this study, student counsellors and psychologists can better develop interventions to support student well-being and academic success in similar crises in the future if such pandemics are yet to be encountered in years to come.

## Methods

### Participants

For this study, an initial sample of 1,200 undergraduate engineering students from Manipal Institute of Technology, India, was recruited.95 participants, however, chose not to provide detailed information, opting out of the study. Consequently, the final analysis was conducted on the remaining 1,105 participants to ensure data quality and integrity. Data was collected for six months, from August 2023 to December 2023. Third-year (2022–2023) and fourth-year (2021–2022) students were only recruited for the survey as they aligned with the inclusion criteria of our study.

The survey was administered using a Google Form in the presence of the researcher to ensure the purpose of the study and its impact on students, thereby bringing clarity and addressing any questions from the participants. Written informed consent was obtained from all participants before the beginning of the survey, and participation was entirely voluntary. Students were assured that no personal identifying information would be collected, nor would their responses be used for purposes other than the study. The data collected from the participants will be stored with the utmost confidentiality, and no financial or academic incentives will be provided.

## Materials

A multidomain self-administered questionnaire ^86^ was used to collect participant data. A multimodal approach was employed to measure the precise measurement of stress among students, as stress is influenced by multiple factors affecting the behaviour and cognition of stressed individuals. ^26^ Table 1 provides the reliability score of Section C and Section D of the questionnaire used in this study. The questionnaire comprised the following structure with four different sections. The overall structure of the multidomain questionnaire used in this study is shown in Figure 1.

A. Section A of the Questionnaire is titled Student Demographic Details (SDD). This section is the general section within any standard questionnaire, which helps to understand the nature of the participants involved in the study. ^27^ Items within this section comprise both categorical and open-ended questions. Gender, Age, and Course year are the details asked for in this section. These variables were considered necessary for a detailed study of stress levels across participants of different genders and age groups.
B. Section B of the Questionnaire is titled Student Environment during ERL (SE_ERL). This component is considered crucial in influencing human stress. ^28^ Student Environment refers to physical, social, and computational environment characteristics. ^26^ Information on the country of residence and the region of stay are the items within the physical environment. Who did they stay with during emergency remote learning, and was their stay with them happy and content? Had any of their close affinity either been infected by COVID-19 or died because of COVID-19? Were the items referring to the social environment. Did the place of stay have a good internet connection was the item referring to the computational environment.
C. Section C of the Questionnaire is titled Perceived Stress during ERL (PSS). This section comprises items from a modified version of the Perceived Stress Scale. ^29^ The modified version of the Perceived Stress Scale (PSS) consists of 14 Likert scale items, changing the contextual meaning by retaining the item’s structural meaning like the original scale. For example, if the scale has the item ‘In the last month, how often have you been upset because of something that happened unexpectedly?’ is now modified into ‘During ERL, how often have you been upset because of something that happened unexpectedly?’. All 14 items within this section are the 5-point Likert questions, with 0 being Never and 4 being rated Very Often. Items numbered 4,5,6,7,8,10, and 13 are negatively rated. The cumulative sum of the scores from all the items is the total score, which is further considered when grading the stress level among the individual. This standard questionnaire was used to assess the intensity of the student’s perceived stress during ERL.
D. Section D of the Questionnaire is titled Perceived Stress of Course Delivery and Learning during ERL (ERL). This section of the questionnaire mainly focuses on the crucial factors that help in the smooth conduction of online or face-to-face learning based on students’ experiences. ^30^ These factors are necessary for developing this questionnaire, as Emergency Remote Learning was a new pedagogy. To aid this pedagogy in processing knowledge, the best factors influencing already existing teaching-learning platforms were of predominant value. A total of 20 Likert-type items are designed with five-point ratings with 0 for Never and 4 for Very Often. The following instruction fields are considered within this section. ^31, 32^
  a. **Learning outcomes:** Items on the difficulty in gaining knowledge, if lack of hands-on experience made them less competent, did they lack problem-solving abilities, and difficulty in participation in class activities are four items that mainly targeted students’ competencies to achieve. ^33^
  b. **Course design and technical issues:** Items on the difficulty in understanding the course structure, if students were annoyed because of submission due dates, assessments, and examinations, and connectivity issues to join live classes were some of the items that were included in this section. These factors, considered as the quality of the learning environment, contribute to the success of the course. ^32^
  c. **Interaction between students and the instructors:** Difficulty contacting instructors, delayed response by instructors, social and emotional connectedness with the instructor, and instructor feedback are the items. Instructors often support students in the learning process, especially on an e-learning platform, helping to build the instructor’s cognitive presence and establish a social relationship with the instructor. ^34^
  d. **Interaction with peer students:** A positive emotional environment enhances positive peer learning. ^35^ Absence of working in groups, feelings of isolation, lack of social and emotional connection with peers, and absence of learning with peers are some of the items within this instruction field.
  e. **Individual learning processes:** Difficulty staying motivated, exhaustion in studies, and disengagement due to lack of co-curricular activities are some of the parameters for which students are expected to be stressed. Motivation has a direct, positive link with stress and a negative association with learning. Students who lack motivation may exhibit higher levels of stress and lower levels of learning satisfaction. ^36^

**Table 1.** Reliability score of Section C and Section D items of the questionnaire.

Questionnaire component	Mean	Standard Deviation	Cronbach’s α	McDonald’s ω
Section C (Perceived Stress during ERL (PSS)	2.0494	0.54178	0.85380	0.86353
Section D (Perceived Stress of Course Delivery and Learning during ERL (ERL)	2.2169	0.62034	0.90584	0.90674

## Procedure

### Recruitment of Students for Data Collection

**Figure 2.**
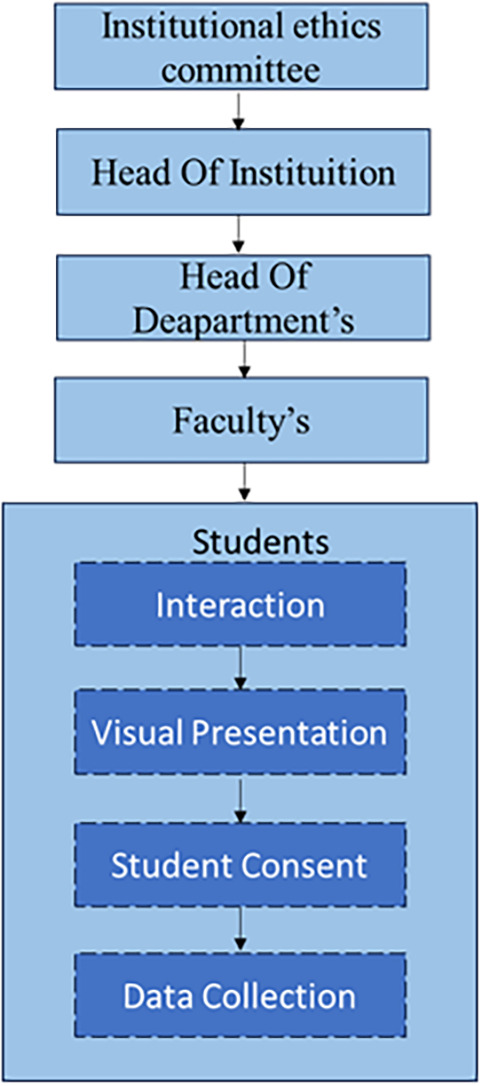
The step-by-step sequence of procedures followed to Recruit Students for Data Collection.

The participants were recruited systematically by getting prior permissions and approvals from the Institutional Ethics Department and the Institutional Heads to interact with students. The steps followed to recruit students are summarised in Figure 2.

1. Step 1: On receiving approval from the Institutional ethics committee to conduct the study, a formal request letter was made to all the heads of the departments across various engineering disciplines to interact with the respective subject faculties of both Final and Pre-final year students to engage their one-hour theory classes.
2. Step 2: A random class across various disciplines was chosen for study as the main interest covered most students to get diverse responses.
3. Step 3: Initially, the study started with Final year students. One hour of the class was engaged to conduct this study. Firstly, the students were introduced to the study’s objectives, purpose, and impact on the students. Second, a visual presentation showing the impact of COVID-19 on university students was displayed for 5 minutes. This was followed by clear instructions about the study, briefing about the structure of the questionnaire, and answering the participants’ queries if they had any.
4. Step 4: Written consent was obtained before they participated in the study. The study was voluntary, and those interested would respond to the questionnaire.
5. Step 5: A digital QR code was displayed, which students had to scan to take the survey. This QR code would open up a Google form displaying their items within the survey. Using the paper and pen method, the online data collection method was chosen to reduce data entry errors and improve data integrity through automated processes. ^37^ The survey neither took any of the personal details of the participants nor their identity recorded, which helped to collect the anonymous data from the beginning of the study. The duration allotted for filling out the questionnaire was 20 minutes.
6. Step 6: The survey was conducted in the presence of the researcher. It was not a fully online survey, as online surveys generally have lower response rates than online data collection ^38^ and higher item omission rates and potential biases due to self-selection. ^39^
7. Step 7: A similar approach was followed to interact with pre-Final year students.

### Data Cleaning and Preliminary Analysis

This section outlines the various strategies for preprocessing the raw survey responses and the methods used to validate the developed tool.

1. Data Cleaning
  a. Item-coding
    i. Items within Section A (Student Demographic Details-SDD) were coded as SDD_1, SDD_2, SDD_3, and SDD_4, respectively.
    ii. Items within Section B (Student Environment during Emergency Remote Learning (SE_ERL)) were coded as SE_ERL1 to SE_ERL7.
    iii. Items within Section C (Perceived Stress during ERL-PSS) were coded as PSS-1 to PSS-14.
    iv. Items within Section D (Student Perception during ERL-ERL) were coded as ERL1 to ERL20.
  b. Coding of Responses
Likert-scale items were coded to enhance the computation of final scores and interpretation of different stress levels. For the Perceived Stress Scale (PSS-1 to PSS-14), the ratings were coded as:0 = Never,1 = Almost Never,2 = Sometimes,3 = Fairly Often,4 = Often. Since items PSS-4, PSS-5, PSS-6, PSS-7, PSS-9, PSS-10, and PSS-13 are negatively worded, their scores are reverse-coded. Similarly, for items ERL1 to ERL20, the following scale was used:0 = Never,1 = Rarely,2 = Sometimes,3 = Fairly Often,4 = Very Often.
  c. Handling of Missing Data
Participants who chose not to participate in the survey were recorded with the response “I disagree.” All such rows were excluded from further analysis and deleted from the dataset.
2. Dataset Validation
  a. Face and Content Validity
The 20 Likert-scale items in Section D of the questionnaire were newly developed, and establishing their validity and reliability was a primary step. A panel of subject matter experts comprising clinical psychologists, student counselors, and language experts with ten years and above years of experience in their respective fields assessed the validity of this section.
  b. Internal Consistency Reliability
The 20 Likert-scale items in Section D of the questionnaire were also checked for reliability using Cronbach’s alpha ^40^ and McDonald’s ω. ^41^ These tests assisted in determining which items should be kept or eliminated. To improve the scale’s dependability, negatively associated items were also thoroughly examined for possible removal.

## Results

### A. Dataset Validation

1. Face Validity
Face validity was found to be satisfactory, with the tool seeming both constructive and relevant after a few items were modified through iterative validity checks. Content validity was evaluated for appropriateness, relevance, and adequacy. The overall S-CVI/Ave was 0.97, and the S-CVI/UA was 0.92, indicating excellent content validity for section D items.
2. Internal Consistency Reliability

The overall Cronbach’s Alpha score was 0.906, which indicates excellent reliability and is much better than the Cronbach Alpha score from Section items of PSS-14, which is 0.85. A detailed description of the reliability analysis of the questionnaire items is shown in Table 1.

### B. Descriptive statistics

The descriptive statistics of the student population who participated in the survey are tabulated in Table 2. Of 1137 student interactions about the study,32 students opted not to participate; hence, the remaining N=1105 students agreed to participate in the survey, and their data was further analyzed for stress level classification.

**Table 2.** Demographic details.

Items	Count of participants (% of count/N)
Gender	
Female	289 (26.2%)
Male	805 (72.9%)
Prefer Not to Say	11 (1.0%)
Age	
19	92 (8.3%)
20	405 (36.7%)
21	503 (45.5%)
22	105 (9.5%)
Course Year	
3rd Year	512 (46.3%)
4th Year	593 (53.7%)
Country of Residence	
Outside India	55 (5.0%)
Within India	1050 (95.0%)
Region	
Rural	88 (8.0%)
Suburban	185 (16.7%)
Urban	832 (75.3%)
Whom did you stay with during ERL?	
Alone	56(5.06%)
Friends	57(5.15%)
Grandparents	158 (14.3%)
Parents	834 (75.5%)
Was your stay happy and comfortable?	
Always	442 (40.0%)
Never	54 (4.9%)
Sometimes	609 (55.1%)
Had any of your relatives/Family members/Friends/Close Affinity got infected with Covid-19?	
No	189 (17.1%)
Yes	916 (82.9%)
Did you had good internet connectivity or access at the place of your stay?	
No	63 (5.7%)
Yes	1042 (94.3%)
Stress levels	
Low Stress	251 (22.7%)
Moderate Stress	292 (26.4%)
High Stress	258 (23.3%)
Very High Stress	304 (27.5%)

This sample population of 1105 students consisted of both 3rd-year and fourth-year students from multiple branches of engineering who were nearly evenly split and primarily male category students (72.9%), with females making up 26.2% and a small proportion (1.0%) who preferred not to disclose their gender. Most participants were 20 and 21, representing 82.2% of the sample. Most students stayed within the country (95.0%), with 75.3% reporting an urban living environment. During this pandemic, 75.5% of the students stayed with their parents, while 14.3% stayed with their grandparents, with a small group living alone (5.06%) or with friends (5.15%). However, only 55.1% reported that their stay was “sometimes” happy and comfortable with their family, while 40.0% indicated it was “always” happy and relaxed. Also, the participants’ stress levels were evenly distributed over the various categories, suggesting varied stress levels among students throughout Emergency Remote Learning.

### C. Student’s responses for the ‘Perceived Stress During ERL’ component of the questionnaire

A modified version of the Perceived Stress Scale (PSS) was used to assess how students perceived ERL as a stress-contributing factor to their overall state of mind. All the 14 items within the scale were modified to fit the context of our study. Each statement was rated as Never,Almost never, sometimes, fairly often, or very often. One sample t-test was conducted to investigate how students’ responses deviated across different items within the scale and confirm whether student responses were fair across all items. The results of the one-sample t-test is tabulated in Table 3.

**Table 3.** Results of One-Sample t-Tests for perceived stress during ERL.

Items	Item-code	M ^a^	SD ^b^	t ^c^	d ^d^	p ^e^
Upset about unexpected things	PSS-1	2.2588	0.87227	86.082	2.5896	<0.001
Inability to control important things	PSS-2	2.4552	1.01083	80.741	2.4289	<0.001
Feeling of nervous and stressed	PSS-3	2.4181	1.07121	75.038	2.2573	<0.001
Irritating life hassles	PSS-4	1.7222	0.91173	62.790	1.8889	<0.001
Coping with important changes	PSS-5	1.7484	0.94027	61.812	1.8595	<0.001
Ability to handle personal problems	PSS-6	1.6154	1.03802	51.731	1.5562	<0.001
The feeling of things going our way	PSS-7	2.1430	0.98052	72.651	2.1856	<0.001
Inability to cope with things	PSS-8	2.0995	0.98496	70.858	2.1316	<0.001
Ability to control irritations	PSS-9	1.7937	0.91730	64.999	1.9554	<0.001
The feeling of being on top of things	PSS-10	2.2317	0.99122	74.842	2.2514	<0.001
Angered by things happening out of control	PSS-11	2.3665	1.02441	76.792	2.3101	<0.001
Thinking about things to accomplish	PSS-12	2.9240	1.05278	92.325	2.7774	<0.001
Ability to control spending of time	PSS-13	1.9964	1.07928	61.488	1.8497	<0.001
The feeling of difficulties piling up	PSS-14	2.1928	1.08178	67.381	2.0270	<0.001

^a^ Mean.

^b^ Standard Deviation.

^c^ t-scores.

^d^ Effect size.

^e^ Probability value (significance level).

**Figure 3.**
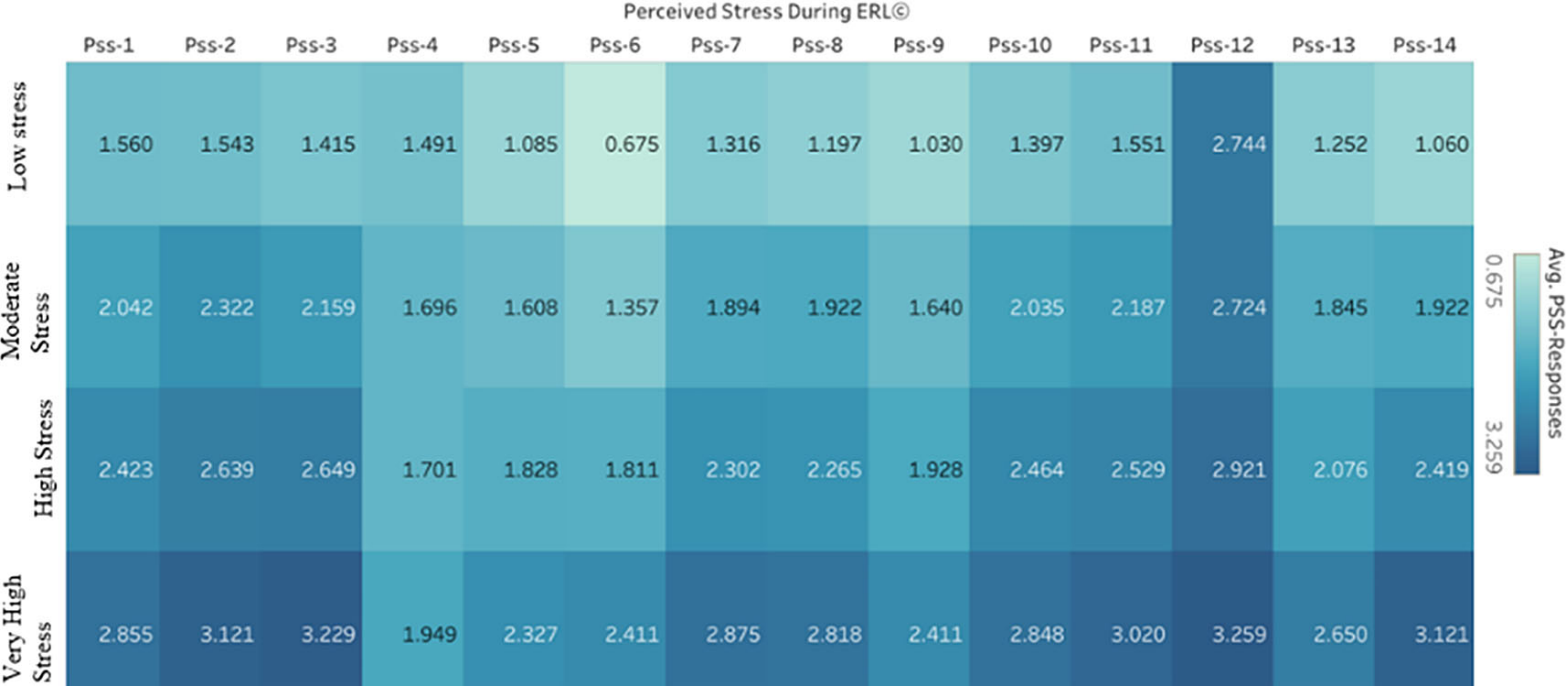
Average Response Rates of Perceived Stress During ERL items across different stress levels.

The students’ stated stress levels varied across the 14 questions, as the mean scores ranged from 1.6154 (PSS-6) to 2.9240 (PSS-12). In comparison, the lower mean for PSS-6 indicates less perceived stress in that context, as students were largely confident about their ability to handle personal problems and did not contribute to stress among them. The relatively higher mean score for PSS-12 suggests that students were particularly stressed about things they had to accomplish. ^42, 43^ The standard deviations show how differently students answered. The item with the most significant impact size in this instance is PSS-12 (d = 2.7774), which lends additional credence to the idea that it is a substantial source of stress for participants. The Average response rates of the participants leading to different stress levels from the Perceived Stress During the ERL component are described in Figure 3.

**Figure 4.**
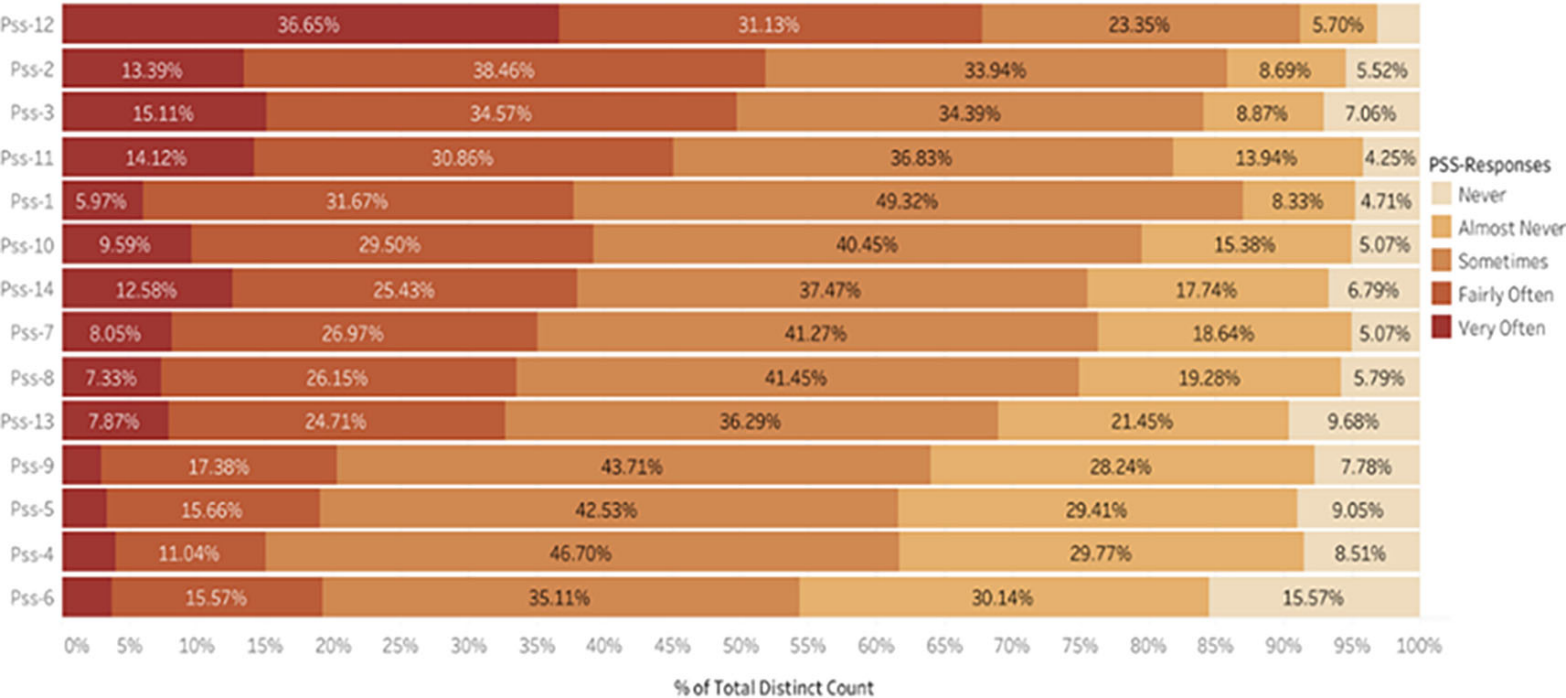
Percentage Distribution of Item Responses of ‘Perceived Stress during ERL (Arranged by Descending Average).

The percentage of the total distinct count of PSS responses is shown in Figure 4. The items are arranged in the descending order of the average responses. While PSS-12 ranks topmost, 36.65% of respondents indicated a response very often, followed by 31.13% selecting it often; this implies that the difficulty of being piled up every passing day was a significant stressor causing stress. ^44^ It also indicates that a major portion of the student population frequently stressed due to this factor and needs a thorough intervention to understand as to what are the difficulties that were being piled up. Moreover, more than 70% of participants experience at least moderate stress related to the PSS-2 item. This reflects that they could not control the crucial things in their life. The pandemic brought many uncertainties for which students were vulnerable as they had to cope with the academics and the situations around them. ^45^ Participants were upset that most of the things were happening unexpectedly, making them stressed, as PSS-1 reflected that over 80% of respondents indicated at least moderate stress due to this. In contrast, just 4.71% were unaffected by these unexpected life outcomes. Similarly, one can observe the variations in stress levels across different items. Overall, the data consistently indicate high levels of stress among participants, particularly indicating that thoughts as to what to accomplish in the future, feelings of failure to control things in life, frequent feelings of stress and nervousness, and persistent getting angry for the things going out of their way made students exhausted and stressful most of the times suggesting the need for targeted mental health resources especially in times of pandemic as students are more exposed to stressful factors which are beyond their control to manage and control all by self.

### D. Student’s responses for Perceived Stress of course delivery and learning during ERL

**Figure 5.**
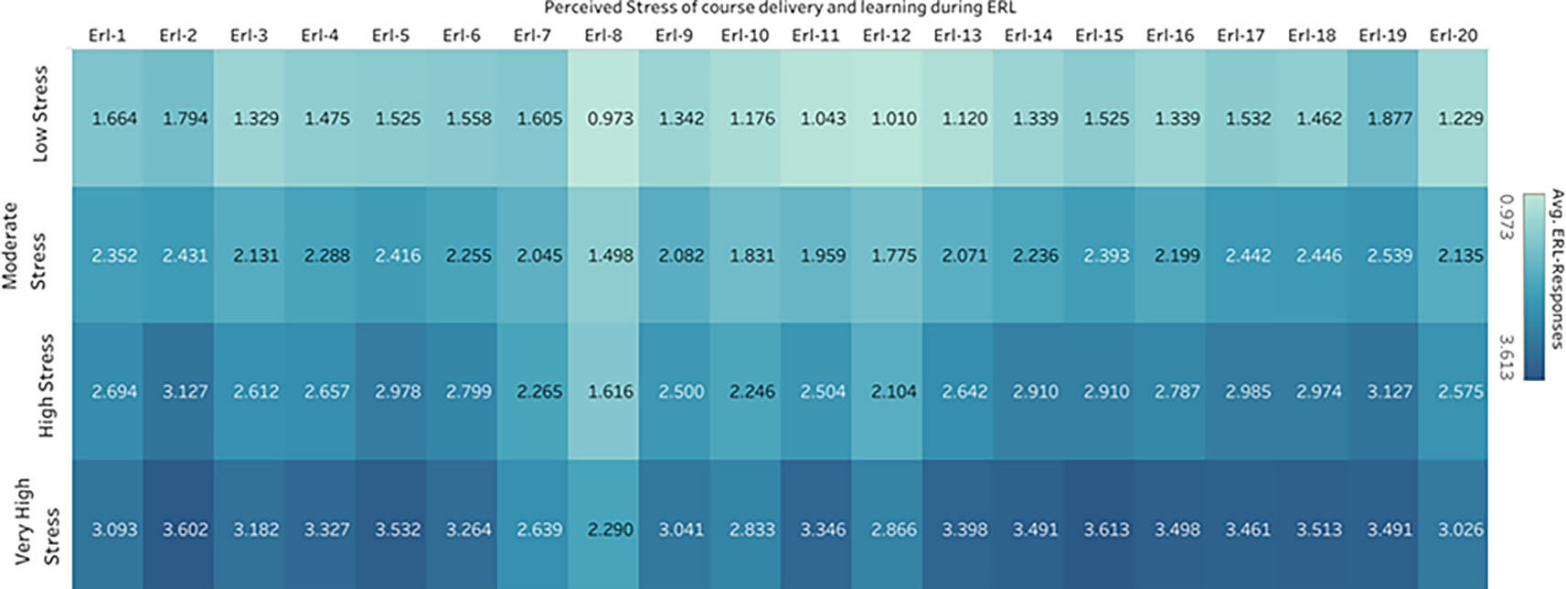
Average Response Rates of Perceived Stress During ERL items across different stress levels.

Apart from the perceived stress of the context of ERL on the participants, the study further explored how ERL as the instruction medium could likely cause stress among subjects. Five different constructs within the instruction medium were further investigated, and 20 Likert scale questions were developed to assess if these could contribute to student stress. Figure 5 shows the Average Response Rates of these 20 likert scale items. One sample t-test was conducted to examine how students’ responses deviated across different items within the scale and confirm whether student responses were fair across all items. The results of the one-sample t-test are tabulated in Table 4.

**Table 4.** Results of One-Sample t-Tests for Perceived Stress of course delivery and learning during ERL.

Items	Item-code	M ^a^	SD ^b^	t ^c^	d ^d^	p ^e^
**Learning Outcomes**						
Knowledge gain	ERL-1	2.4281	1.1820	68.282	2.0541	<0.001
Hands-on experience	ERL-2	2.7113	1.1590	77.766	2.3394	<0.001
Problem-solving abilities	ERL-3	2.2851	1.1362	66.854	2.0112	<0.001
Participation in class activities	ERL-4	2.4090	1.2601	63.549	1.9117	<0.001
**Course Design and technical issues**						
Ongoing course structure	ERL-5	2.5810	1.1691	73.385	2.2076	<0.001
Submission due dates	ERL-6	2.4425	1.2455	65.190	1.9611	<0.001
Assessments and Examination	ERL-7	2.1231	1.1014	64.076	1.9276	<0.001
Connectivity issues for Online classes	ERL-8	1.5765	1.1748	44.606	1.3419	<0.001
**Interaction between students and instructors**						
Contacting instructors for help	ERL-9	2.2154	1.1806	62.378	1.8765	<0.001
Response to queries by instructors	ERL-10	1.9973	1.1952	55.551	1.6711	<0.001
Social and Emotional Connectedness with Instructors	ERL-11	2.1792	1.2650	57.266	1.7227	<0.001
Feedback from the instructors	ERL-12	1.9122	1.1525	55.152	1.6591	<0.001
**Interaction with peer students**						
Working in groups	ERL-13	2.2733	1.2588	60.030	1.8059	<0.001
One-to-one interaction	ERL-14	2.4606	1.2455	65.674	1.9757	<0.001
Social and Emotional connection among peers	ERL-15	2.5792	1.2119	70.748	2.1283	<0.001
Positive learning environment with peers	ERL-16	2.4235	1.1970	67.304	2.0247	<0.001
**Individual learning processes**						
Staying self motivated	ERL-17	2.5738	1.2047	71.018	2.1364	<0.001
Self-study of subjects	ERL-18	2.5656	1.2487	68.297	2.0546	<0.001
Extra and co-curricular activities	ERL-19	2.7330	1.1246	80.785	2.4302	<0.001
ERL as the main stressor	ERL-20	2.2118	1.1093	66.280	1.9939	<0.001

^a^ Mean.

^b^ Standard Deviation.

^c^ t-scores.

^d^ Effect size.

^e^ Probability value (significance level).

Table 4 Results of One-Sample t-Tests for Perceived Stress of course delivery and learning during ERL

1. **Learning outcomes:** Items numbered ERL-1-ERL-4 are the items that mainly focus on the learning outcome of the course taught during ERL. While the ERL-1 item shows a moderate average score with high statistical significance(M=2.428,d=2.0541), ERL-2 is the item with the highest mean(M=2.7113,d=2.3394) among these four items, ERL-2 suggests that students felt particularly strong about this aspect of their experience were lack of hands-on experience have made them feel less competent and has indirectly contributed as a stressor. ^46, 47^ At the same time, items ERL-3 and ERL-4 show a similar trend with moderate mean and vital significance.
2. **Course design and technical issues:** ERL-5-ERL-8 items focus on course design and any technical issues during ERL. Among these items, participants strongly agree that the ERL-5 has a relatively high mean score and moderate variation in the responses among the participants(M=2.5810, SD = 1.1691). This represents that they found it difficult to follow the ongoing course structure during ERL, which has contributed mainly as the stressor causing stress among students during ERL. However, with a mean of M=2.4425 and SD =1.2455, the ERL-6 item shows moderate agreement among the participants with more variability in student responses. Meanwhile, the ERL-8 item had a lower mean score (M=1.5765), suggesting that students’ perceptions of this item were generally less intense. In comparison to earlier items, the effect size is lower (d = 1.3419), and the t-score is 44.606. This indicates a significantly weaker but still considerable agreement. As a result, it is crucial to create support networks and course arrangements that heavily prioritize the welfare of students. ^48^
3. **Interaction between students and instructors:** Items ERL-9-ERL-12 focused on the interaction between students and instructors. ERL-9 and ERL-11(M=-2.2154, SD=1.1806 andM=2.179, SD=1.2650) suggest a moderate level of student perception about contacting the instructor for help was a stressor ^49^ at the time and a large effect size(d= 1.8765) indicates a strong association between this item and students’ perceptions during ERL. Lack of social and emotional connectedness has contributed to the stress factor among students and is also considered significantly as contributing to stress among students, ^50^ with the effect size d=1.7227, indicating that the item had a substantial impact on the students’ experience.
4. **Interaction with peer students:** ERL-13-ERL-16 are the items focused on interacting with peers. Lack of one-to-one interaction among the peers, item ERL14(M=2.4606, d=1.9757), reflects more substantial student agreement. ^51^ At the same time, ERL 15(M=2.5792, d=2.1283) had a significant mean and effect size, which indicates that this item was one of the most agreed-upon by students, which signifies that lack of social and mutual connection from peers significantly contributed to their stress levels. Also, ERL16 (M=2.4235, d=2.0247) indicates a strong agreement among the participants that the absence of a positive learning environment with peers has demotivated their learning process. ^52^
5. **Individual learning processes:** ERL-17-ERL-20 are the items focused on the Individual learning processes: ERL-17(M=2.5738, d=2.1364), ERL-18(M= 2.5656, d=2.0546) and ERL-19(M=2.7330, d=2.4302) demonstrated a strong agreement among participants which indicate they have contributed as a stressor during ERL. Difficulty in staying motivated, self-learning, and the feeling of disengagement ^53– 55^ from extra and co-curricular activities are the factors that have led to various levels of stress among participants during ERL.

**Figure 6.**
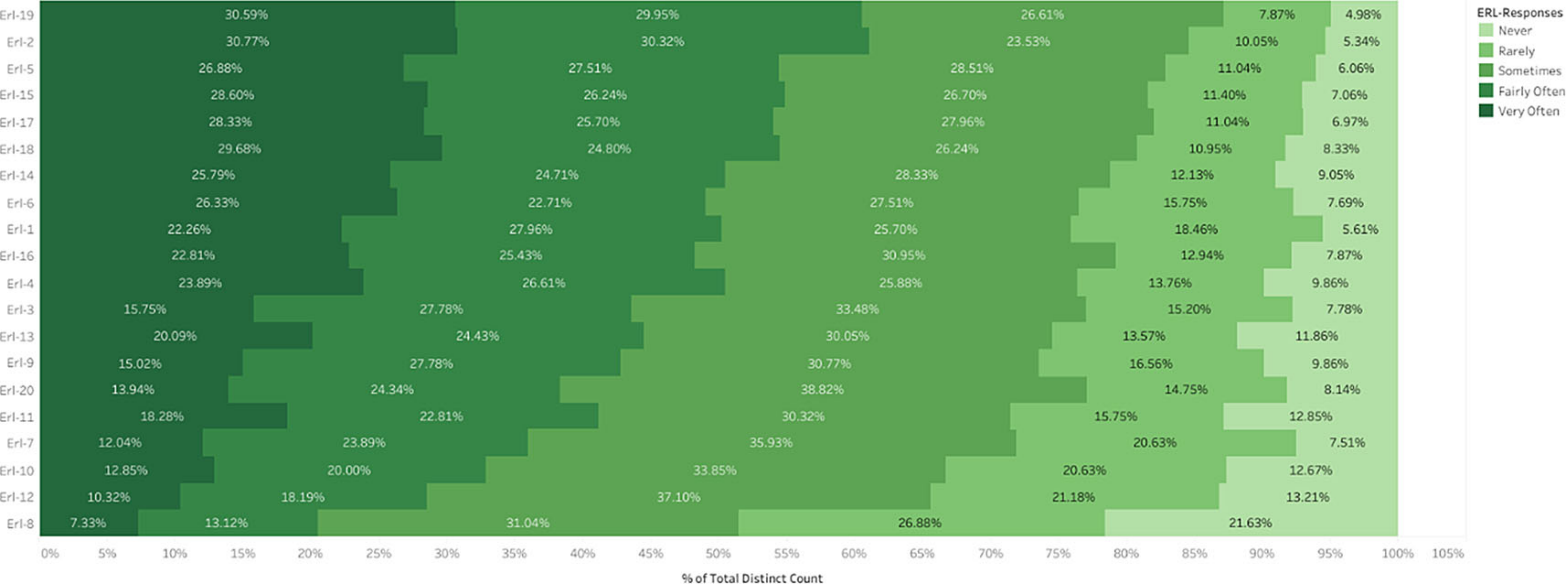
Percentage Distribution of Item Responses of ‘Perceived Stress of Course Delivery and Learning during ERL’ (Arranged by Descending Average).

Figure 6 represents the percentage distribution of item responses arranged in the decreasing order of the average responses. ERL-19 has the highest average response, indicating a lack of active participation in extra and co-curricular activities. This leads to 30.59% of responses to the highest level of 4, indicating many students experienced stress due to this item. Following this is the ERL-2, suggesting a lack of hands-on experience, which resulted in 30.77% of the students experiencing very often the factor of being stressed. A substantial 28.60% of respondents chose the highest option of 4 for item ERL15, indicating a lack of mutual social and emotional connection among peers made them feel left out, resulting in 28.60% of respondents selecting option 4. This also acted as a significant stressor among students. A similar distribution of student responses across items within Perceived stress of course delivery during ERL. According to the rankings of the ERL items, many students chose the most significant levels of felt stress, making lack of participation in co-curricular activities and lack of hands-on experience the most important stressors. In contrast, items at the lower end indicate a less stressful relationship, such as ERL-8(internet connectivity to attend online classes) and ERL-12(lack of adequate and timely feedback).

### E. Correlation Analysis of Perceived Stress during ERL items and the Total PSS score

**Figure 7.**
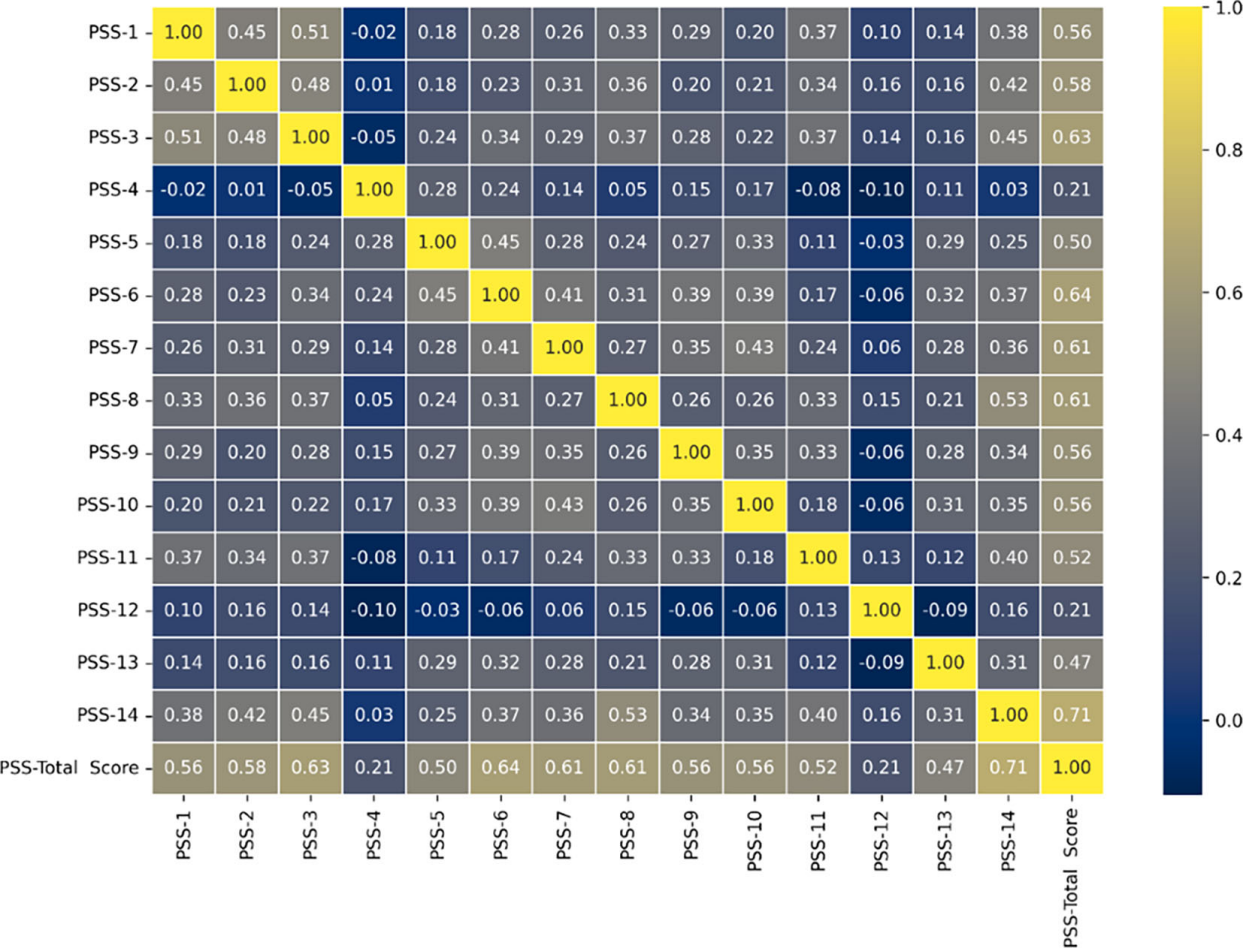
Spearman Correlation matrix of Perceived Stress during ERL items and the Total PSS score.

The Spearman correlation coefficient ^56^ was calculated between the Perceived stress during ERL items and the total PSS score to observe how each item correlates with the overall perceived stress level. PSS-14 (0.71), PSS-6 (0.64), PSS-3 (0.63), PSS-7 (0.61), and PSS-8 (0.61) have relatively high correlation coefficients (above 0.60), meaning responses to these items have contributed significantly to the overall perception of stress among participants. Spearman Correlation matrix of Perceived Stress during ERL items and the Total PSS score is shown in Figure 7.

These items reflect the feeling of difficulties being piled up every passing day, lack of confidence to handle their problems, often feelings of nervousness and stress, the feeling that things were not going their way, and lack of ability to cope with ongoing things around during the phase of a pandemic have positively contributed to the increased level of stress among students. Whereas items like PSS-2 (0.58), PSS-1 (0.56), PSS-9 (0.56), PSS-4 (0.21), and PSS-12 (0.21) have a moderate to low correlation with the total PSS-score. Therefore, items with strong correlation values indicate critical indicators of stress levels that would need further interventions during the pandemic to take care of the mental state of the students experiencing stress because of these.

### F. Correlation Analysis of Perceived Stress of Course Delivery and learning during ERL items and its Final Score

Based on the Spearman correlation analysis, the items reflecting the learning outcomes from the course have a varying coefficient score, out of which items ERL-2(0.65) and ERL-3(0.64) address lack of hands-on experience and lack of problem-solving abilities caused them stress. Whereas items about course design and any other technical issues, ERL-5(0.68) identified as difficulty to follow the then ongoing structure of the course is said to contribute to the stress positively. Items that relate to the interaction between the students and the instructors ERL-11(0.70) and ERL-12(0.61), which reflect the lack of social and emotional connectedness and adequate and timely instructor feedback, have added to their stress levels.

**Figure 8.**
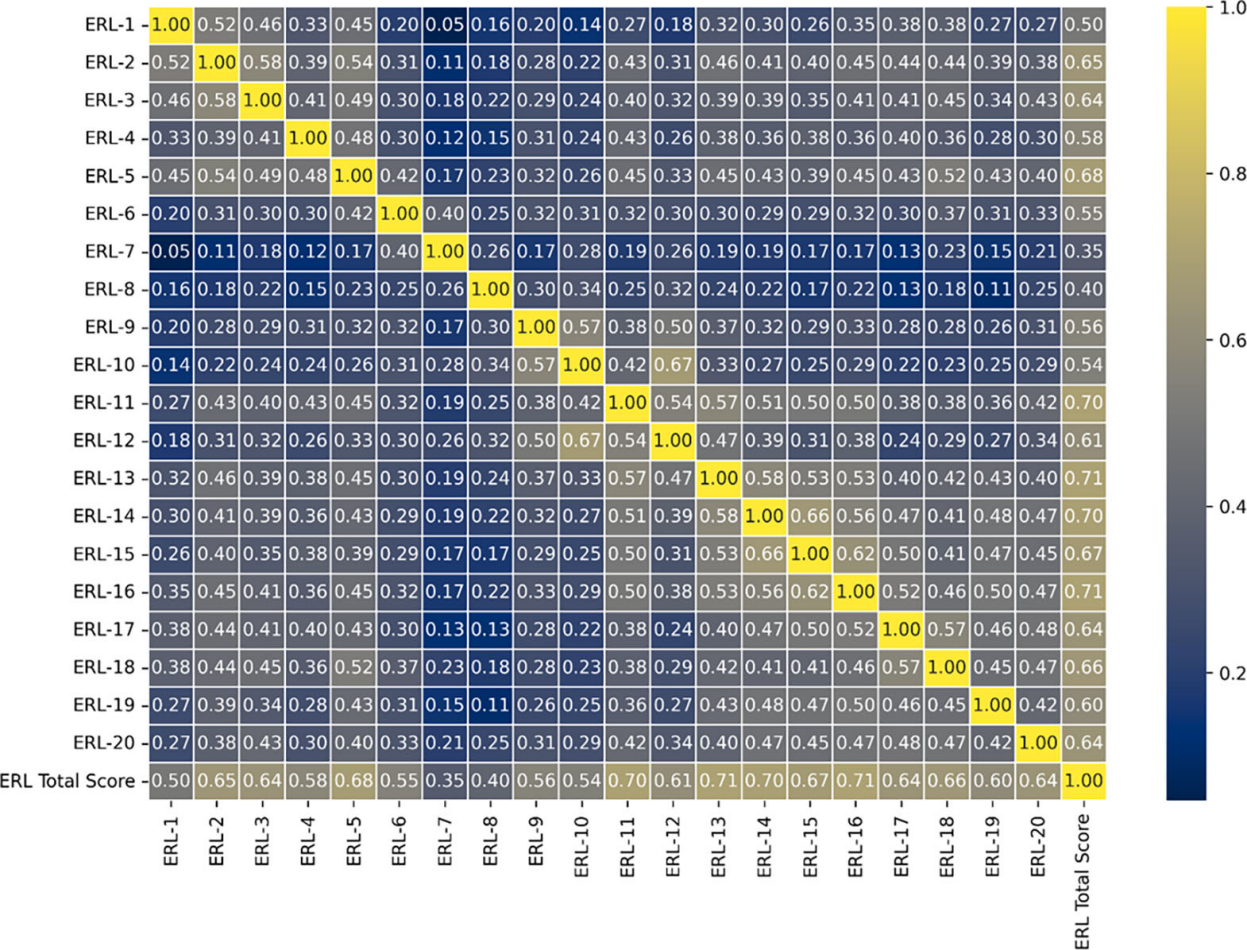
Spearman Correlation matrix of Perceived Stress of Course Delivery and Learning during ERL and Total ERL score.

Items numbered ERL-13-ERL-16 reflect pivotal stressors focusing on the interaction among peers, and the items from ERL-17-ERL-20 focus on individual learning processes, which have majorly contributed to the total stress during ERL. Figure 8 is the correlation matrix using the Spearman coefficient technique that captures the most impactful stressors, which likely align with the broader experiences of students during this period. ERL-13 and ERL-16 are, in particular, the potent stress contributors during remote learning.

For researchers and educators, these findings highlight the areas where students are most vulnerable to stress. Interventions in these areas during the Emergency could focus on working on these factors, thereby providing better academic support by providing better access to resources and fostering social and emotional connectivity among instructors and peers.

## Data visualizations

### A. Student demographic details

**Figure 9.**
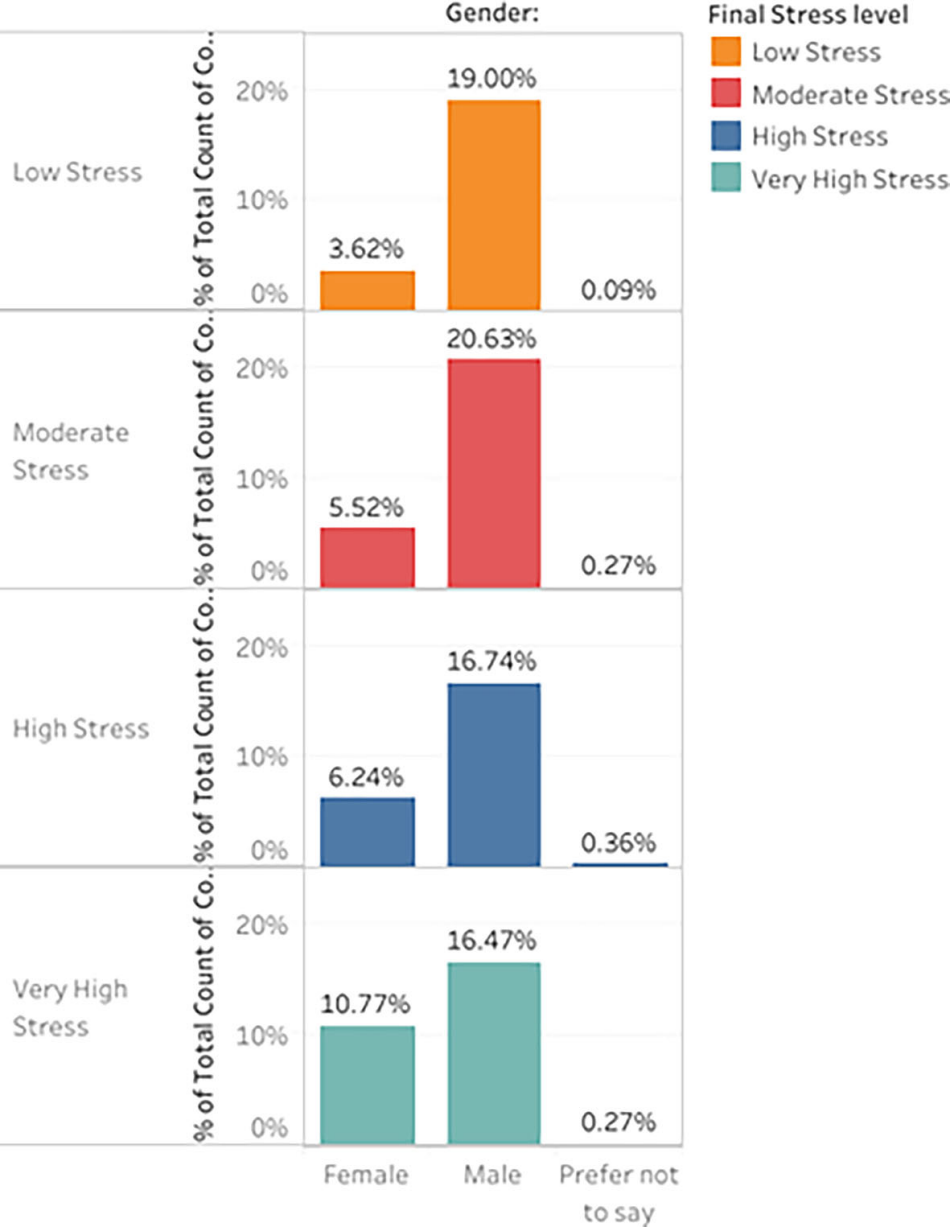
Percentage Distribution of participant responses for a) Stress levels Vs Gender b) Stress levels Vs Age.

This study also incorporated the visualization of final stress levels to that of different variables with the questionnaire tool. Students’ demographics mainly help to understand which gender was more exposed to stress and how different age groups perceived the situation as stressful. Figure 9(a) shows the visualization made for the distribution of final stress vs. gender. In contrast, Figure 9(b) shows the visualization for the distribution of final stress among different age groups of participants.

Most males (16.742%) reported high-stress levels compared to females (6.244%). On the other hand, Very high stress was higher in females (10.769%) than in males (16.471%). ^57, 58^ Among different age groups of participants in the study, those aged 21 (10.588%) reported the highest high stress, ^59^ followed by those aged 20 (8.597%) also exhibited the highest high stress.

### B. Student environment during ERL

1. *Distribution of stress levels across various Regions within and outside the country.*

**Figure 10.**
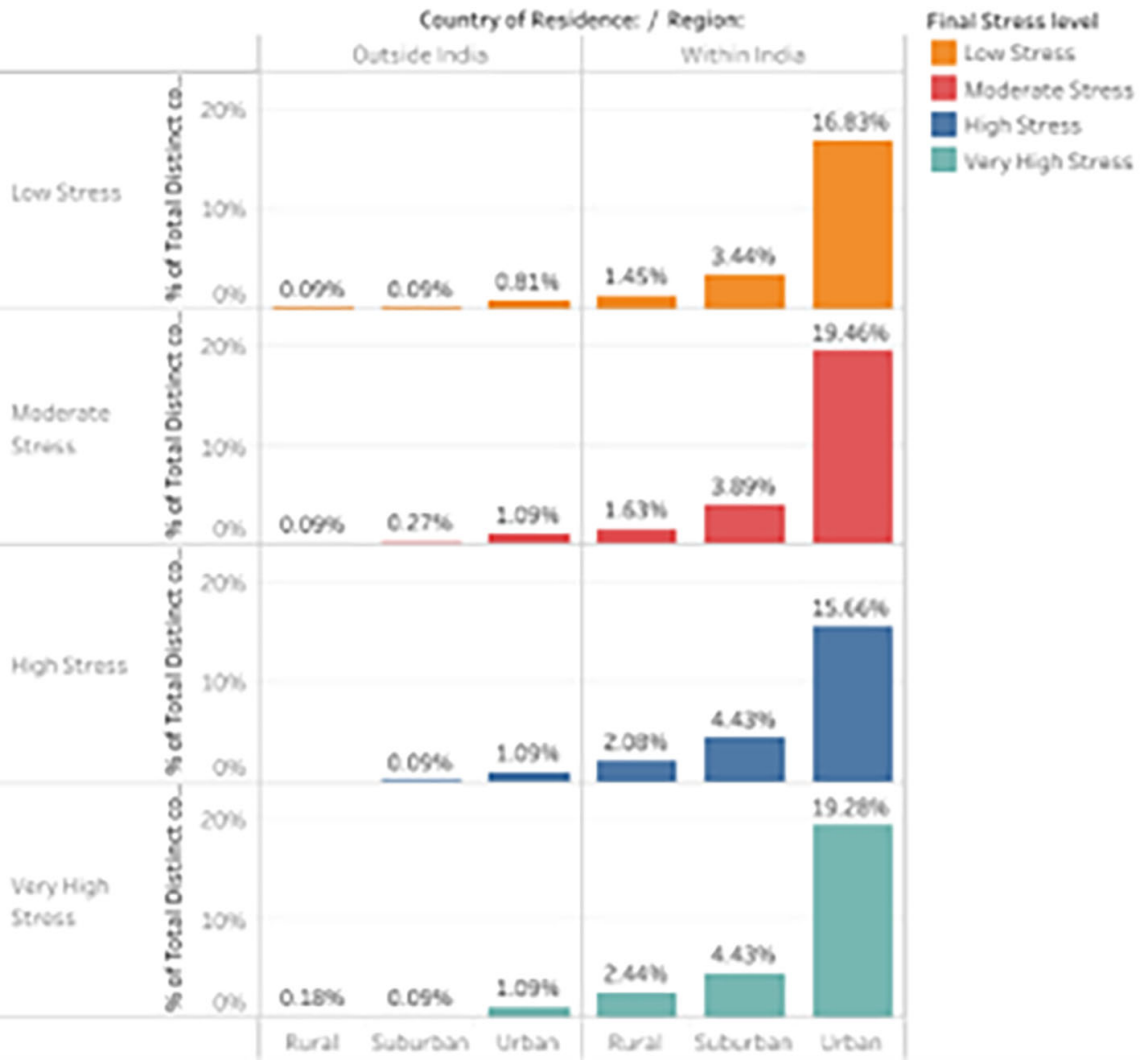
Percentage Distribution of participant responses across various Regions within and outside the country.

The distribution of stress levels across several environmental variables during ERL is visualized, which facilitates an examination of the differences in stress levels between pupils living in India and those outside the country, further classified into rural, suburban, and urban areas. Understanding stress patterns across these environmental variables facilitates better insights into how geographic and regional contexts affect perceived stress during Emergency Remote Learning. ^60^ Figure 10 is a bar plot representing the distribution of stress levels among participants from different regions within and outside the country.

Students living outside of India often have lower stress counts (1.086%) across all stress levels, suggesting that they may have had better access to coping strategies or better learning settings during Emergency Remote Learning. Comparatively, students in India, especially those living in cities, reported much higher levels of stress (15.656%), and 19.276% reported extremely high levels of stress. This indicates that urban students in India were more likely to experience stress, ^61^ possibly due to environmental or academic pressures that later worsened due to remote learning during the pandemic. Surprisingly, participants from rural areas both inside and outside of India reported the least amount of stress. In India, only 2.443% of rural students reported significantly high-stress levels, demonstrating that the environment was comfortable and pleasant and was not as impacted by the pandemic’s stresses. Participants in suburban regions reported moderate stress, with 4.434% expressing very high stress and 4.434% reporting high stress. Although suburban locations generally balance urban and rural settings, there was still a noticeable stress level among the students here, albeit not as much as in metropolitan areas. ^62^

2. *Distribution of stress levels based on whom the participants stayed with during ERL*

**Figure 11.**
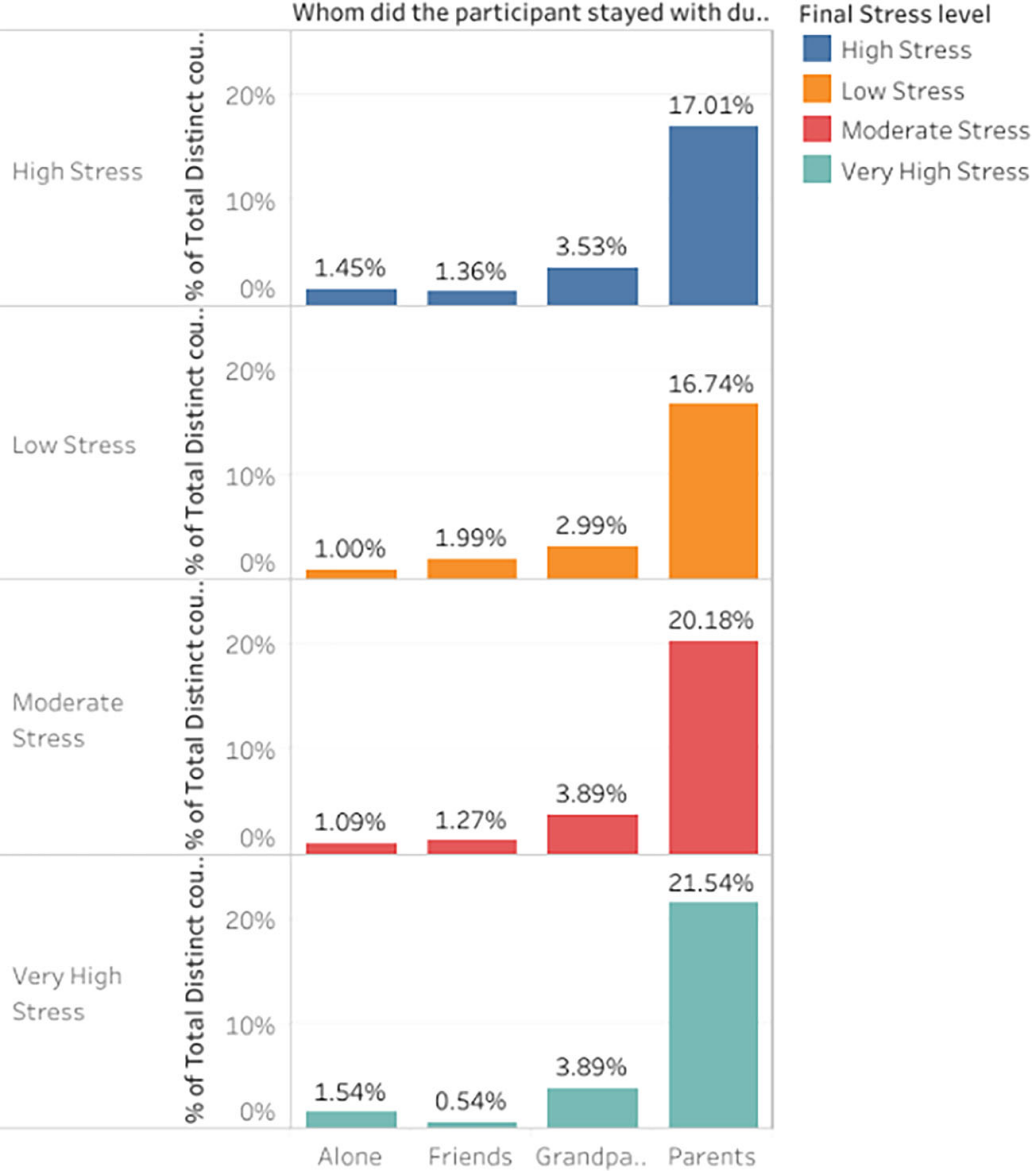
Percentage distribution of participant responses based on whom the participants stayed with during ERL.

This visualization investigates how students’ stress levels vary according to living conditions. The barplot representation in Figure 11 evaluates the differences in stress levels among students who lived with parents, grandparents, friends, or alone. Understanding this distribution is crucial to identifying which groups were most affected by stress during ERL.

The group of students who stayed with their parents was the largest regarding diversity in stress levels. Notably, 17.014% of students expressed significant stress, and 21.538% indicated high stress. On the other hand, 16.742% of students who lived with their parents reported feeling less stressed. These results suggest that while some students found stability in living with their parents during the pandemic, others may have experienced pressures associated with family, ^63^ resulting in different stress levels. 3.529% of the participants reported feeling highly stressed, while 3.891% said that staying with grandparents caused them to feel extremely stressed. Those who lived with senior family members during the pandemic may have been more concerned about their health, adding to their stress levels. On the other hand, students who lived with friends displayed a more evenly distributed stress pattern, with significant percentages across all stress levels. Considerable stress was experienced by students living alone, ^64^ especially very high stress (1.448%) and high stress (1.267%). This shows that stress levels during ERL may have been increased by isolation, as anxiety and mental strain may have increased due to a lack of environmental support. ^65^

3. *Distribution of stress levels based on whether their stay was happy with the person they stayed with during ERL.*

**Figure 12.**
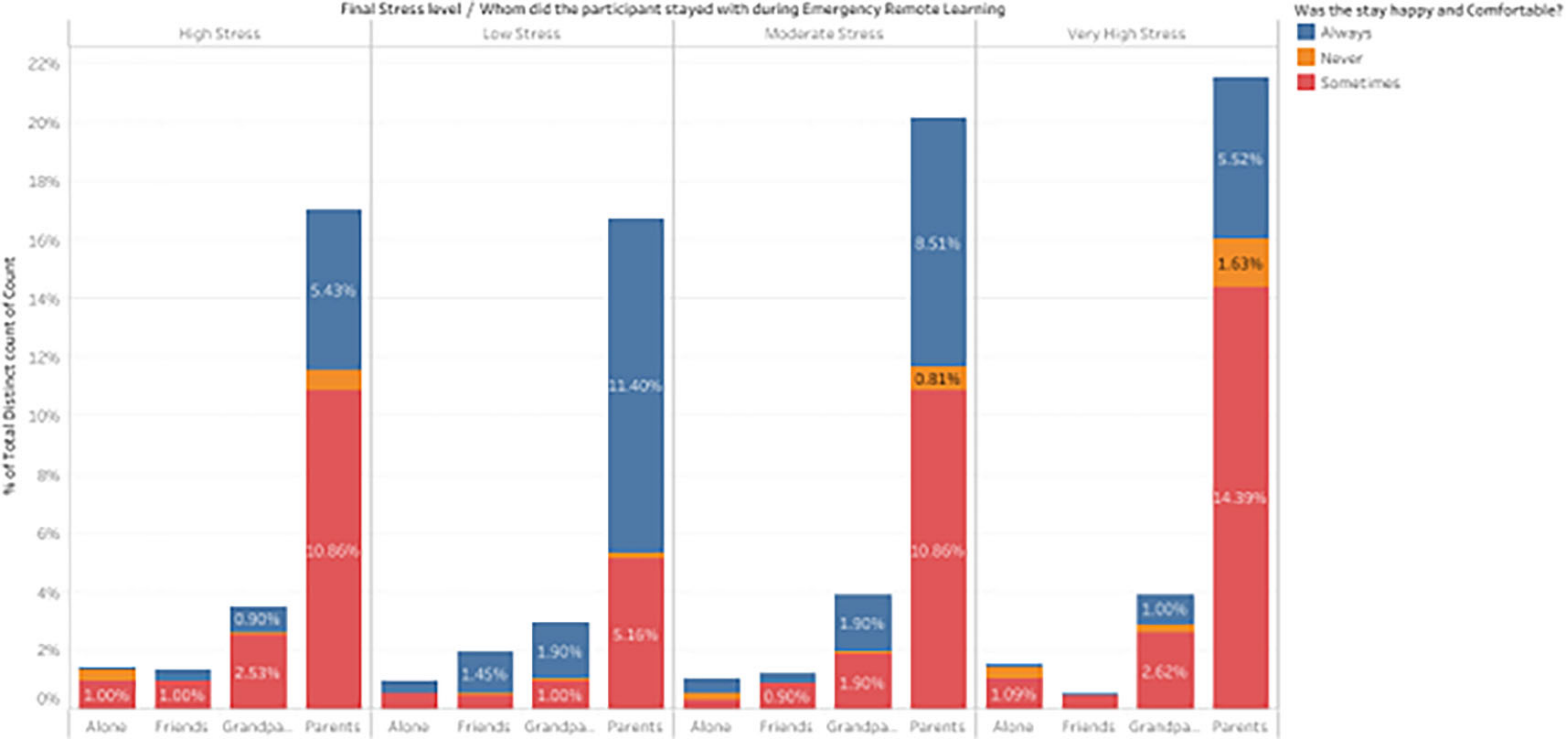
Percentage distribution of participant responses based on whether their stay was happy with the person they stayed with during ERL.

This visualization helps to understand the relationship between whom students stayed with during Emergency Remote Learning (ERL), their perception of how happy and comfortable the stay was, and the final stress levels (low, moderate, high, and very high) will help us to understand what mattered the most to students during ERL. Figure 12 describes whether the stay was happy with whoever they stayed with during ERL.

Students who reported being “always” happy during their stay and were living with their parents had the highest percentage of low stress (11.40%). This pattern implies that parents’ emotional support was a significant factor in stress reduction during ERL. Similarly, people who visited their grandparents and reported being “always” happy also had less stress (1.90%). When a student’s stay was “sometimes” enjoyable, they experienced moderate stress more frequently (10.86%). However, less in number than those remaining with parents, similar patterns are observed among those staying with grandparents. In contrast to students who reported “sometimes” or “never” joyful stays, those who stayed alone or with friends showed lower stress percentages when their stay was “always” cheerful and fewer reports of severe stress. The most impacted group of students who had extremely high levels of stress stayed with their parents and described their stay as “sometimes” cheerful (14.39%), indicating that not all forms of parental support helped reduce stress. Those staying alone also reported very high stress (0.99%) and described their stay as “sometimes” enjoyable.

4. *Distribution of stress levels based on whether COVID-19 infected anybody in their close affinity?*

**Figure 13.**
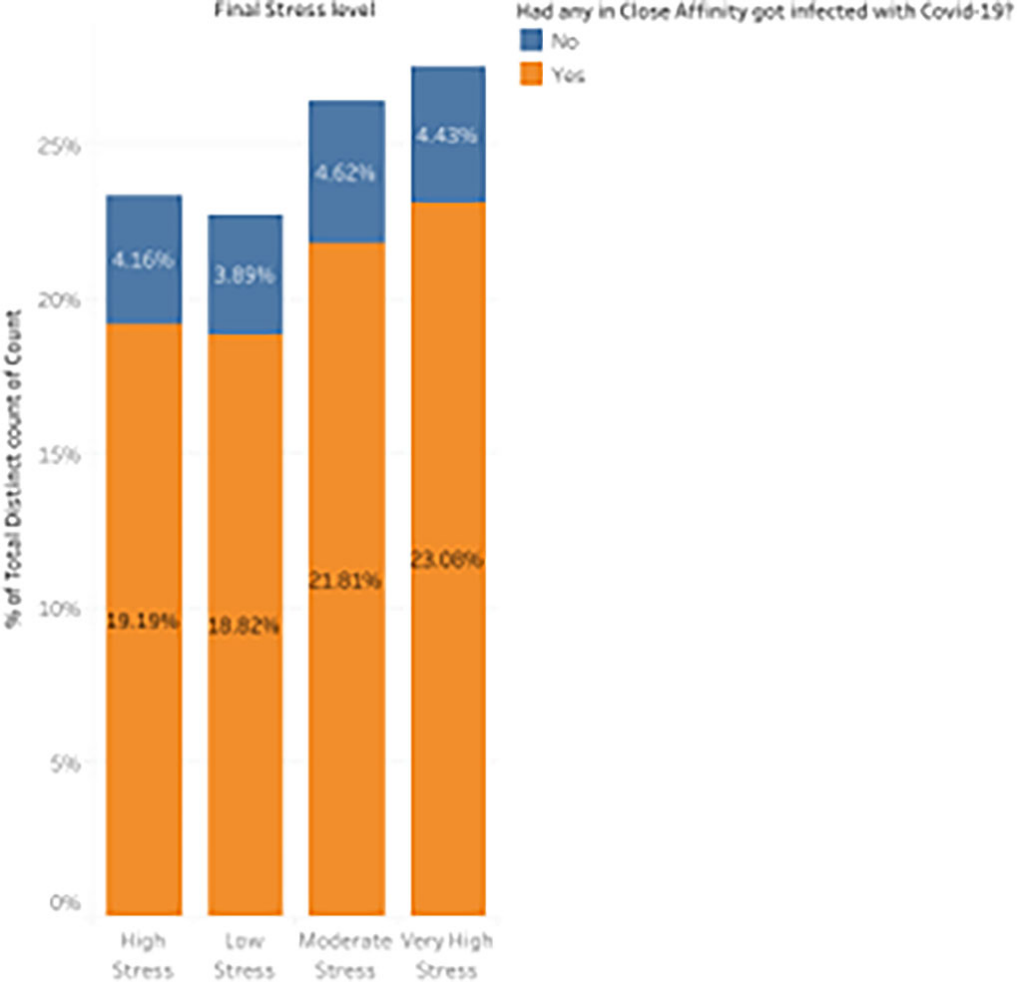
Percentage distribution of participant responses based on whether COVID-19 infected anybody in their close affinity.

This visualization examines how close affinities with COVID-19 infection affect students’ stress levels, thoroughly investigating stress distribution throughout low to extremely high-categories. Figure 13 represents a distribution plot that draws attention to the psychological toll the pandemic took on students, emphasizing how external health concerns impacted their stress levels.

19.19% of students with infected close affinities reported feeling stressed out. This is a notable rise in stress compared to individuals without an infected family (4.16%), suggesting that worry or dread for the well-being of loved ones played a significant role in their experience. ^66, 67^ Remarkably, 18.82% of students said they felt no concern even if they knew someone afflicted. This suggests that while many students experienced stress, some were resilient or less impacted by the pandemic’s direct effects on their families. When students’ close affinities were infected, the percentage of them experiencing moderate stress rose to 21.81%. This shows that the circumstance had a mild but discernible effect on the student’s mental health, even though the stress was not excessively high. Significantly, 23.08% of participants who had close relatives afflicted by the virus indicated highly high-stress levels. This dramatic rise highlights the tremendous anxiety and psychological strain students have when COVID-19 directly impacts friends or family members.

5. *Distribution of stress levels based on if anybody of their close affinity died due to COVID-19.*

**Figure 14.**
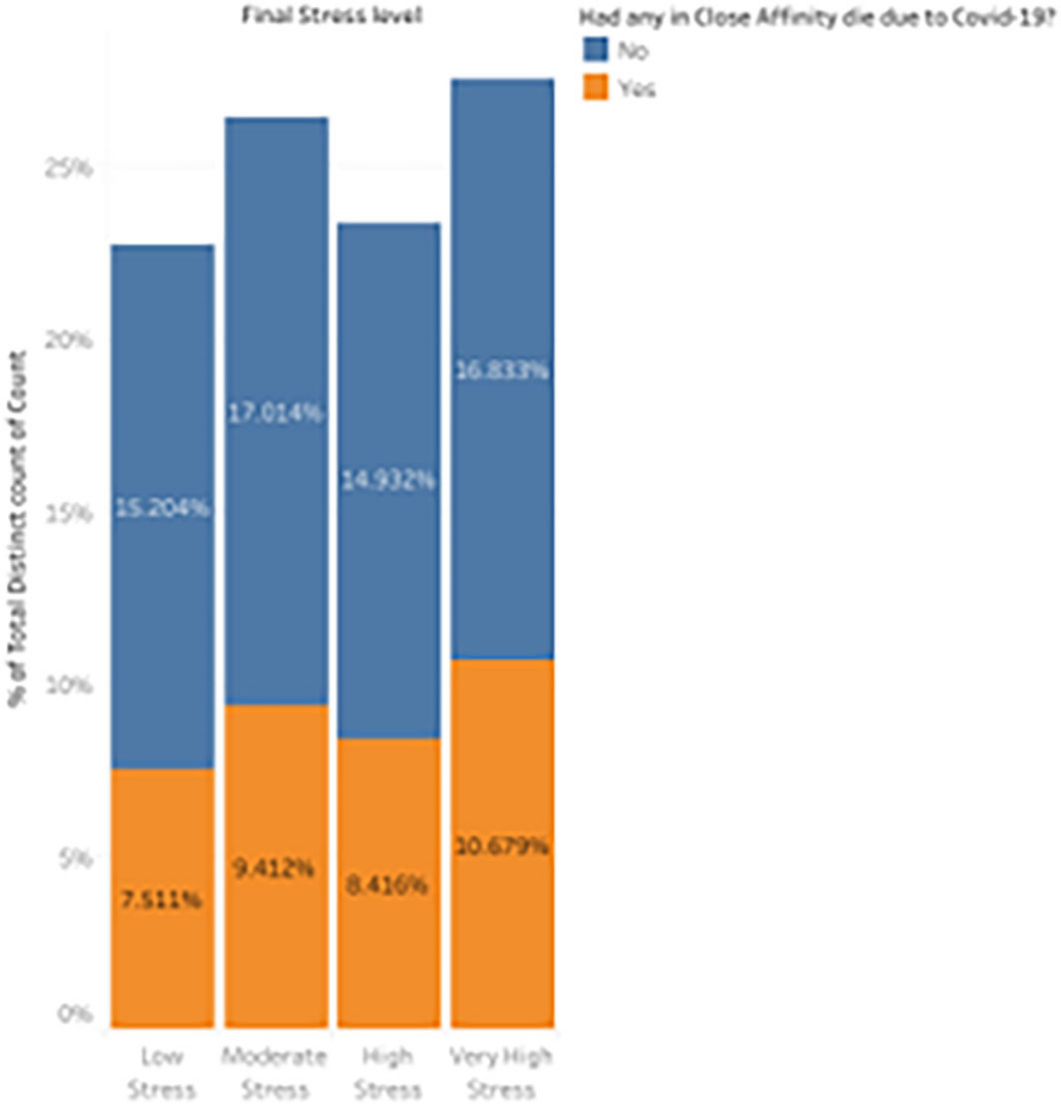
Percentage distribution of participant responses based on if anybody of their close affinity died due to COVID-19.

The loss of close affinities during the COVID-19 pandemic took an emotional toll never seen before, which might exacerbate stress in students already managing the rigors of remote learning and the unpredictability of the world health emergency. ^68, 69^ This visualization looks at how students’ stress levels were affected by losing friends, family, or other close relations, with an emphasis on how grief increased psychological strain. Figure 14 provides a distribution plot depicting the spread of low to extremely high-stress levels, underlining the significant emotional toll inflicted by such losses during the pandemic. This analysis clarifies the pandemic’s broader effects on mental health, especially concerning stress and grief.

8.416% of students who had a death in their close affinity reported considerable stress, compared to 14.932% of students who did not lose anyone close to them. This demonstrates a moderate difference between the two groups of students: those who lost a close relative experienced lower levels of extreme stress. Of the students who did not encounter a death, 15.204% reported having low-stress levels, compared to 7.511% of those who did. This implies that pupils who experienced the death of a close relative experienced a discernible decline in low-stress levels. ^70^ Compared to 9.412% of students who experienced a death, a more significant percentage of students (17.014%) who did not lose anyone reported experiencing considerable stress. This discrepancy suggests that students who did not experience a direct loss were more likely to experience moderate stress. The biggest difference may be seen in the very high-stress category, where 10.679% of students who experienced a death reported experiencing very high stress, compared to 16.833% of students who did not lose someone. This shows that individuals who experienced a loss were somewhat less likely to experience extreme stress, which may indicate that other coping strategies were used in the wake of the catastrophe.

6. *Distribution of stress levels based on whether good internet connectivity existed?*

**Figure 15.**
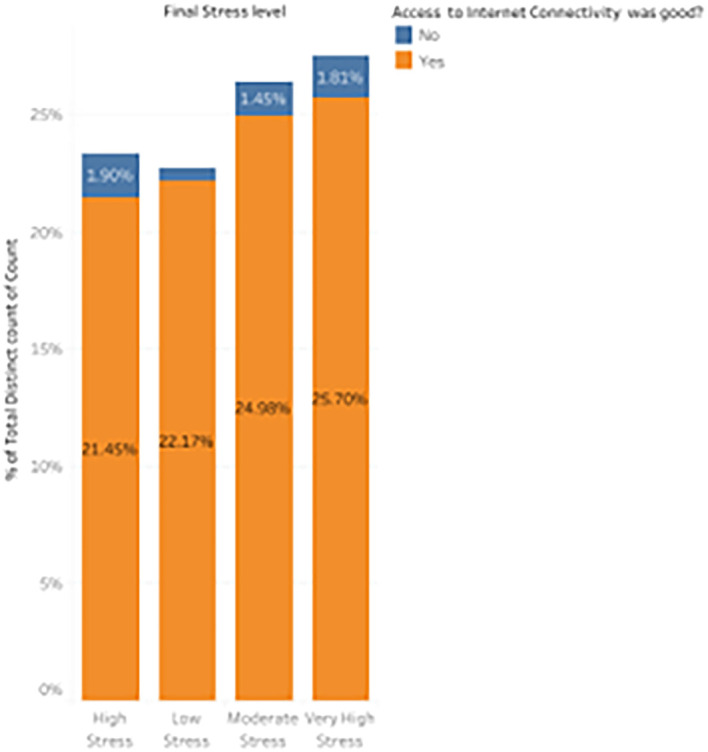
Percentage distribution of participant responses based on whether good internet connectivity existed.

During Emergency Remote Learning (ERL), one crucial aspect affecting students’ stress levels was the availability of reliable internet connectivity. The abrupt transition to online learning increased the need for dependable internet ^71, 72^ since any outage could negatively impact students’ well-being and academic achievement. This visual aid investigates the relationship between stress levels, from very low to very high, and the availability or lack of reliable internet connectivity. A distribution map illustrating the psychological toll associated with technical issues during ERL is shown in Figure 15. This analysis clarifies how, during the pandemic, restricted internet access contributed an additional layer of stress to an already difficult academic and emotional environment.

The data shows that 21.45% of students with good internet access experienced high stress, while a small proportion of students without internet access (1.9%) also reported high stress. This suggests that even students with good connectivity were immune to high stress, highlighting the multifaceted nature of stress contributors. For low stress, 22.17% of students with reliable internet access reported experiencing low stress, compared to only 0.54% without connectivity. This suggests that students with stable internet tended to experience significantly less stress, likely due to fewer academic disruptions. Moderate stress levels follow a similar trend, with 24.98% of students with good internet access reporting moderate stress, while only 1.45% of those without access experienced moderate stress. Finally, 25.70% of students with good connectivity fell into this category for very high stress, compared to 1.81% of students without access. From these observations, we can infer that internet connectivity was not a definitive predictor of low stress; instead, prolonged screen time would also have contributed to variation in stress levels among students. ^73, 74^ Most students with good internet access still reported moderate to very high-stress levels. This indicates that while technical barriers certainly contributed to stress, other factors, such as the emotional and academic challenges of the pandemic, were also significant stressors. The findings suggest that ensuring access to stable internet is necessary but insufficient for managing stress during remote learning. Further support systems addressing emotional well-being and academic pressures are equally essential. ^73, 75, 76^ This distribution emphasizes the need to consider broader solutions beyond technological fixes to help students cope with stress in remote learning contexts.

7. *Distribution of responses to Perceived Stress During ERL items across different stress levels.*

**Figure 16.**
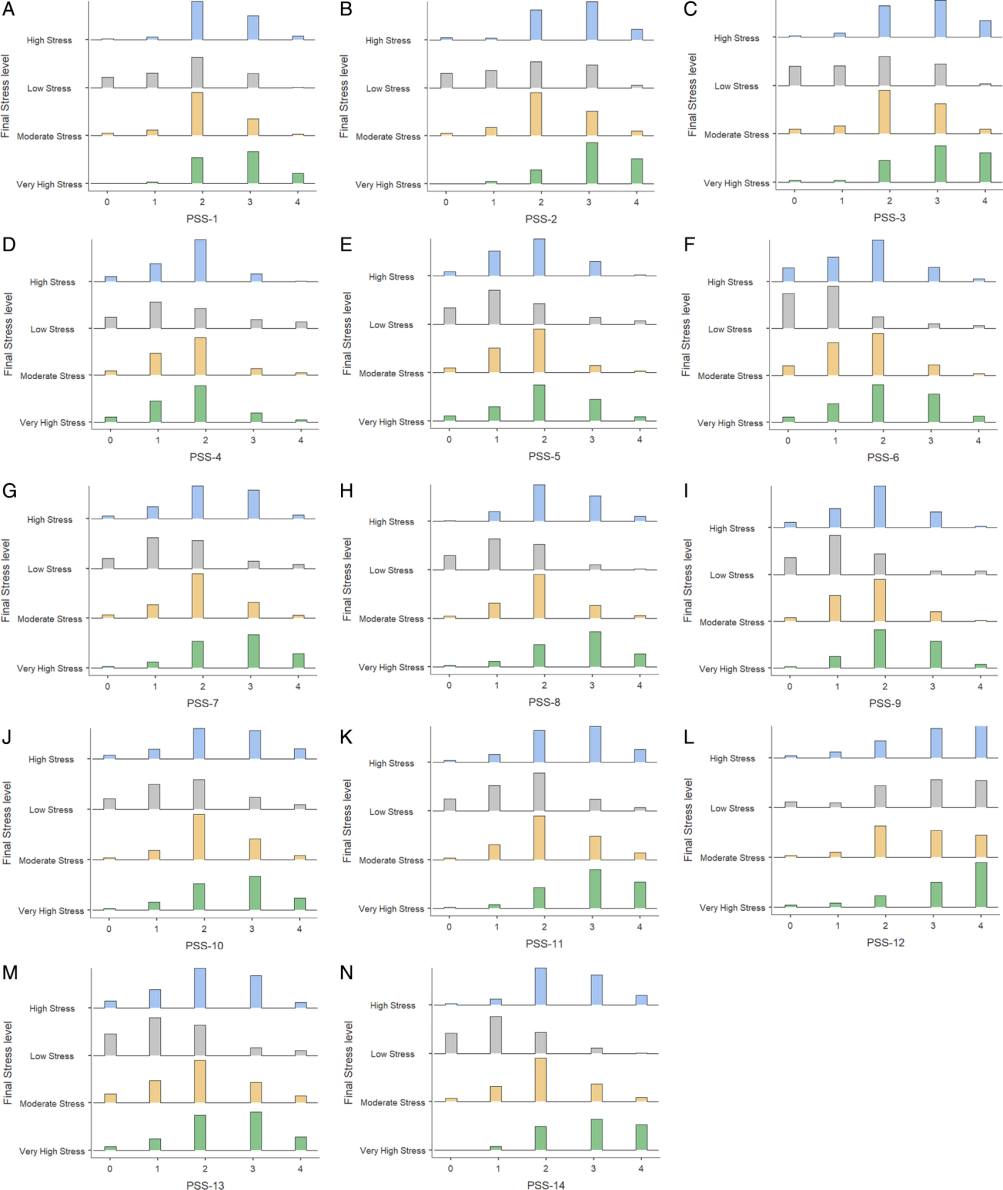
(A-N). Distribution of Student responses for all Perceived Stress During ERL items.

This visualization investigates how various facets of students’ perceptions of stress led to a range of stress intensity, from minimal to extremely high. It does this by examining the distribution of responses to the Perceived Stress During ERL items and emphasizing the vital role that some stressors—like persistent ideas about impossible tasks and helplessness—played in students’ psychological health. A distribution plot is shown in Figure 16(A-N), which highlights how these internal stressors—such as the inability to manage time or accumulating challenges—shaped the students’ stress responses and offers a detailed examination of the underlying causes of moderate and high-stress levels.

The most frequent contributor to very high stress was PSS-12, with 14.12% of students who scored a four on this item (indicating very often). This item likely measures the students frequently used to think about what they have to accomplish, leading to very high stress. Items like PSS-3, PSS-7, PSS-10, PSS-13, and PSS-14 also had higher frequencies of responses leading to very high stress, with percentages ranging from 8.95% to 12.67%. These items likely tap into nervousness, the feeling of things not going their way, the inability to control how they spend time, and difficulties piling up every passing day. Meanwhile, PSS-2 showed 10.77% to 13.67% in the high-stress category. This suggests that moderate feelings of stress were elevated when students could not control the important things in their lives, leading to frequent frustration or challenges. PSS-5, PSS-9, and PSS-11 (11.04% - 14.03%)indicate that students would effectively manage the critical changes that were occurring, could control irritation in their life, and control the things that were beyond their control respectively experience moderate stress but don’t feel entirely out of control. PSS-6 reported low stress, indicating they could handle their problems and did not contribute much to their stress levels.

8. *Distribution of responses to Perceived Stress of course delivery and learning during ERL items*

**Figure 17.**
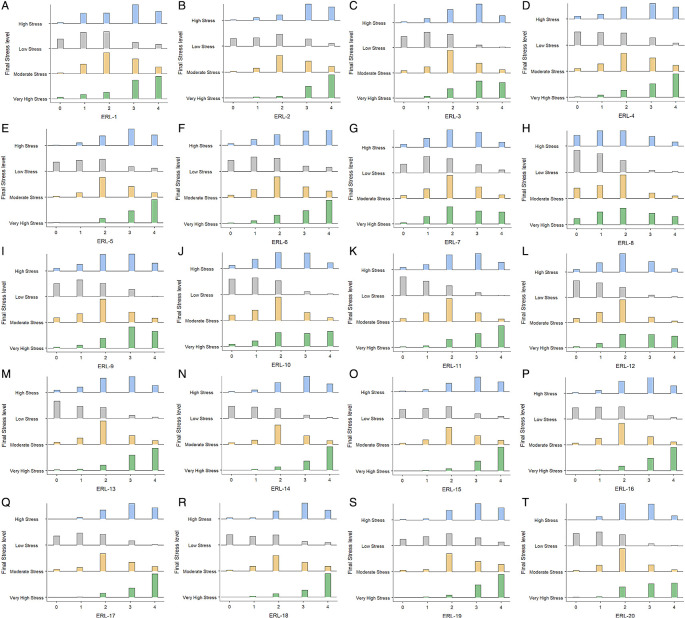
(A-T). Distribution of Student responses to Perceived Stress of course delivery and learning during ERL items.

This visualization examines how various Emergency Remote Learning (ERL) components impacted students’ stress levels, offering a detailed breakdown of responses across low to very high-stress categories. This component, consisting of 20 items within the questionnaire, shed light on students’ perceptions of the challenges and demands they faced during this transition, such as technological issues, lack of engagement, and difficulties adapting to the new learning format. Figure 17(A-T) provides a distribution plot highlighting how these instructional challenges contributed to rising stress levels, revealing how workload, support, and technological barriers affected students’ overall well-being during ERL.

Participants reported that item ERL-20 had the highest percentage of responses, indicating that ERL contributed to their overall stressed state of mind, leading to very high stress (10.045%). With 7.964% responses indicating very high stress, ERL-4 likely captures students’ stress in acquiring sound and adequate problem-solving abilities. Problem-solving, which often requires direct feedback, guidance, and collaborative discussions, might have been challenging in the remote setting. The absence of real-time support and hands-on experiences may have intensified these difficulties, contributing to the heightened stress. Also contributing significantly to very high stress (10.679%), the ERL-3 relates to uncomfortable participating in class activities. Virtual participation lacks the structured environment of physical classrooms, leading to issues like reduced engagement, difficulties in communication, and hesitation in speaking up during online sessions. These challenges may have created a barrier to active involvement, further elevating stress levels. ERL-2(9.593%), ERL-1(4.525%), and ERL-5(5.430%) showed a notable frequency in the high-stress category, indicating a lack of hands-on experience, difficulty in gaining enough knowledge about subjects taught and difficulty to follow the course structure during ERL predominantly lead to perceived challenges within the learning environment, during remote learning. Moderate stress was associated with difficulty contacting the instructors (ERL-9, 12.217%) and lack of social and emotional connectedness with instructors (ERL-11, 13.575%), indicating a transitional period for students that involved various struggles but not overwhelming distress. Whereas the items contributing to low-stress levels reflect positive experiences or supportive conditions during remote learning, suggesting that submission due dates contributed less to their overall stress levels.

## Discussions

The present study aimed to evaluate and visualize the dataset of survey responses to assess how students perceived stress during Emergency Remote Learning (ERL). The survey comprised a multidomain questionnaire with various components. The modified version of an established Perceived stress scale (PSS) and a newly developed set of 20 items focusing on ERL-specific factors majorly lead to assessing stressors contributing to stress. Our results highlight several key trends. First, as the PSS scores indicate, most students reported moderate to high-stress levels during ERL. This finding aligns with previous research suggesting that the transition to online learning during the COVID-19 pandemic has been a significant stressor for students worldwide. ^77, 78^ Additionally, responses to the ERL items reveal that specific factors related to ERL, such as technological barriers, social isolation, and perceived learning effectiveness, were strongly associated with increased stress levels. ^79– 81^

The comparison between the PSS scores and ERL results provides valuable insights. While the PSS captured general stress, the ERL items addressed specific challenges posed by ERL, offering a more nuanced understanding of student stressors. For instance, items like ERL-8 revealed that students who reported higher isolation levels were likelier to exhibit elevated stress scores. This finding is consistent with previous studies highlighting social isolation as a critical factor influencing mental health during remote learning. ^82, 83^ Interestingly, items related to students’ perceptions of their internet connectivity contributing to their overall stress state of mind had moderate stress levels, suggesting that while internet and other technological barriers did contribute to their psychologically distressed state of mind, they were less prominent than factors like social isolation or technological challenges. This might reflect students’ adaptability to technological glitches compared to social and environmental stressors. This finding contrasts with earlier research, ^84^ which suggested that academic pressure was the predominant stressor during online learning.

Moreover, the significant correlation between environmental factors (e.g., ERL-7: “Comfort of Stay During ERL”) and stress levels further supports the notion that stress during ERL was solely driven by academic factors, environmental, social and not majorly on technological issues. This underscores the need for future studies to examine how these factors contributing to student stress can be avoided and work on alternate solutions to avoid students experiencing stress. The results of this study have several practical implications for educators, policymakers, and institutions. Our findings, particularly those related to ERL, suggest that students expect a good social and emotional connectedness with the instructors and their peers, which otherwise have significantly contributed to higher stress levels, which may negatively impact their learning outcomes. Second, our study highlights the importance of addressing students’ social needs. Institutions should consider developing online communities and support systems to reduce feelings of isolation among students. Frequent online interactions among remote learners could also help mitigate the mental health challenges identified in this study. Lastly, developing a context-specific questionnaire like ours is a valuable approach to assessing student stress more accurately. Traditional tools like the PSS are helpful for general stress measurement but may overlook stressors unique to situations like Emergency Remote Learning.

### Limitations of the dataset

While this study provides meaningful insights, there are several limitations to consider. First, the sample was limited to students from a specific geographic region, which may limit the generalizability of the results to other populations. Second, the cross-sectional nature of the data collection means that causality cannot be inferred; longitudinal studies would be needed to assess how stress levels evolve over time. Additionally, the reliance on self-reported data may introduce biases, as students may underreport or overreport their stress levels due to recall bias or the time lapse between the main pandemic event and the data collection process.

#### Ethics and consent

The recruitment of subjects began only after receiving ethical clearance from the Institutional Ethics Committee at Kasturba Medical College & Kasturba Hospital, Manipal (IEC1:411/2022) on (14/12/2022) and registering with the Clinical Trials Registry-India (CTRI) with registration number CTRI/2023/06/053972). Written informed consent was obtained from all participants before the beginning of the survey, and participation was entirely voluntary.

## Data Availability

Figshare: Questionnaire Dataset on Perceived Stress of Students During Emergency Remote Learning https://doi.org/10.6084/m9.figshare.27617160.v3 ^85^ The project contains the following underlying data:
• Combined_DataFile.csv: This is the raw data file consisting of all the 1105 responses collected during the survey. Data are available under the terms of the Creative Commons Attribution 4.0 International license (CC-BY 4.0). Perceived Stress of Students During Emergency Remote Learning (PSSDERL)Questionnaire”. figshare, 20-Nov-2024, doi: 10.6084/m9.figshare.27860535.v1. ^86^ The project contains the following extended data:
• Perceived Stress of Students During Emergency Remote Learning Data are available under the terms of the Creative Commons Attribution 4.0 International license (CC-BY 4.0).
