# Supplementary material for: A Questionnaire Dataset on Perceived Stress in Indian Higher Education Students during Emergency Remote Learning
Source: F1000Res. 2025 Jan 7;14:40. [Version 1] doi: 10.12688/f1000research.159192.1 (PMC12375186; doi:10.12688/f1000research.159192.1)
Supplement: Peer review history

# Reviewer report: Reviewer response for version 1

**DOI:** 10.5256/f1000research.174884.r402110

**Published:** 2025-08-23

**Authors:** Arvind Kumar

**Affiliations:** 1 ECE, National Institute of Technology Kurukshetra, Kurukshetra, Haryana, India; 2 NIT Kurushetra, Haryana, India

This manuscript presents a well-structured and timely dataset study that explores perceived stress levels among Indian engineering students during the COVID-19-driven shift to Emergency Remote Learning (ERL). Using a multidomain questionnaire that includes demographic factors, a contextually adapted PSS-14 scale, and a newly developed 20-item ERL-specific stress scale, the authors collected responses from 1,105 students across academic years and disciplines. The study demonstrates strong internal consistency (Cronbach’s α = 0.91), employs appropriate descriptive and correlational analyses, and is well-supported with clear visualizations. The open-access dataset is ethically sound, well-documented, making it a valuable resource for future research on student mental health, academic resilience, and remote learning. While the study's strength lies in its contextual relevance and methodological clarity, future research could enhance generalizability through multi-institutional sampling, apply factor analysis for deeper construct validation, and integrate longitudinal or multimodal data to explore evolving stress dynamics.

 Authors need to answer the following questions:

 1. How can the validity of the newly developed ERL questionnaire (section D) be established beyond face and content validity?

 2. What was the rationale for modifying the PSS-14 scale instead of using it in its

 original form? Did authors test if the modification affected psychometric properties?

 3. Is author’s dataset reusable and FAIR-compliant (Findable, Accessible, Interoperable,

 and Reusable)?

Are sufficient details of methods and materials provided to allow replication by others?

Yes

Is the rationale for creating the dataset(s) clearly described?

Yes

Are the datasets clearly presented in a useable and accessible format?

Yes

Are the protocols appropriate and is the work technically sound?

Yes

Reviewer Expertise:

Communication Systems

I confirm that I have read this submission and believe that I have an appropriate level of expertise to confirm that it is of an acceptable scientific standard.

# Reviewer report: Reviewer response for version 1

**DOI:** 10.5256/f1000research.174884.r402111

**Published:** 2025-08-13

**Authors:** Pramod Kumar

**Affiliations:** 1 Central University of Karnataka, Kalaburagi, Karnataka, India

This article is on a dataset that documents the development and open release of a novel dataset capturing student-reported stress levels during the transition to Emergency Remote Learning (ERL) within the Indian higher education system. The structured format, comprehensive documentation, and contextual specificity make the dataset suitable for further comparative, predictive, and policy-driven research in academic stress and education technology. The dataset was collected using a structured tool that combines established and custom-designed survey items. The authors employ both item-wise and total-score-based analyses, complemented by correlation analysis to identify trends in stress indicators. Importantly, the dataset is shared under open-access conditions with appropriate ethical safeguards. This contribution is particularly useful for researchers interested in the intersection of technology, pedagogy, and student mental health in the post-pandemic context.

**The following concerns should be addressed by the authors**

1. How were the final stress levels (low, moderate, high, very high) classified from the PSS and ERL scores? Were empirical cutoffs used?
2. This study is limited to students from one institute. To what extent are the findings generalizable to broader student populations in India?
3. Was there any psychological risk to students while responding to items related to death, infection, or stress? Were any safeguards offered?

Are sufficient details of methods and materials provided to allow replication by others?

Yes

Is the rationale for creating the dataset(s) clearly described?

Yes

Are the datasets clearly presented in a useable and accessible format?

Yes

Are the protocols appropriate and is the work technically sound?

Yes

Reviewer Expertise:

Stress management, heart rate variability, Communication

I confirm that I have read this submission and believe that I have an appropriate level of expertise to confirm that it is of an acceptable scientific standard.
